# Feasibility of tissue re-biopsy in non-small cell lung cancers resistant to previous epidermal growth factor receptor tyrosine kinase inhibitor therapies

**DOI:** 10.1186/s12890-017-0514-3

**Published:** 2017-12-06

**Authors:** Sakurako Uozu, Kazuyoshi Imaizumi, Teppei Yamaguchi, Yasuhiro Goto, Kenji Kawada, Tomoyuki Minezawa, Takuya Okamura, Ken Akao, Masamichi Hayashi, Sumito Isogai, Mitsushi Okazawa, Naozumi Hashimoto, Yoshinori Hasegawa

**Affiliations:** 1 Department of Respiratory Medicine, Fujita Health University School of Medicine, 1-98 Dengakugakubo, Kutsukake-cho, Toyoake, Aichi 470-1192 Japan; 2 Department of Clinical Oncology, Fujita Health University Hospital, 1-98 Dengakugakubo, Kutsukake-cho, Toyoake, Aichi 470-1192 Japan; 3 Department of Respiratory Medicine, Daiyukai General Hospital, 1-9-9 Sakura, Ichinomiya, Aichi 491-8551 Japan; 4 Department of Respiratory Medicine, Nagoya University Graduate School of Medicine, 65 Tsurumai-cho, Showa-ku, Nagoya, Aichi 466-8560 Japan

**Keywords:** Re-biopsy, Molecular targeted therapy, EGFR-TKI, Metastasis, Retrospective analysis, Bronchoscopy, CT guided needle biopsy

## Abstract

**Background:**

When epidermal growth factor receptor (EGFR) gene mutation-positive non-small cell lung cancer (NSCLC) acquires resistance to the initial tyrosine kinase inhibitor (TKI) treatment, reassessing the tumor DNA by re-biopsy is essential for further treatment selection. However, the process of TKI-sensitive tumor re-progression and whether re-biopsy is possible in all cases of acquired resistance to EGFR-TKI remain unclear.

**Methods:**

We retrospectively analyzed data from 69 consecutive patients with EGFR gene mutation-positive advanced NSCLC who had been treated with EGFR-TKI and exhibited disease relapse after initial disease remission. The relapsing lesions were identified at the time of RECIST-progressive disease (PD) and clinical-PD (when the attending physician judged the patient as clinically relapsing and stopped EGFR-TKI therapy). We determined the potential re-biopsy methods for each relapsing lesion and evaluated their feasibility according to difficulty and invasiveness criteria as follows: category A, accessible by conventional biopsy techniques; category B, difficult (but possible) to biopsy and accessible with invasive methods; and category C, extremely difficult to biopsy or inaccessible without using highly invasive methods, including surgical biopsy.

**Results:**

The total feasibility rate of re-biopsy (category A or B) was 68% at RECIST-PD and 84% at clinical-PD, and the most common accessible relapsing lesions were primary tumors at RECIST-PD and pleural effusion at clinical-PD. All relapsing lesions at primary sites (categories A and B) were assessed as having the potential for re-biopsy. However, re-biopsy for metastasis was assessed as difficult in a substantial proportion of the study population (42 and 20% category C at RECIST-PD and clinical-PD, respectively).

**Conclusions:**

Re-biopsy of relapsing disease is feasible in many cases, although it may present difficulties in cases with, e.g., metastatic relapsing lesions. To facilitate treatment strategies in NSCLC patients with relapse after EGFR-TKI therapy, re-biopsy should be standardized with the use of simpler and more reliable methods.

## Background

Lung cancer is the leading cause of cancer death globally. Up to 85% of lung cancers are non-small cell lung cancer (NSCLC), and they occur most frequently in an advanced stage of adenocarcinoma [1]. Epidermal growth factor receptor (EGFR)-activating mutation is one of the most common oncogenic driver mutations, especially in eastern Asia (including Japan). EGFR tyrosine kinase inhibitor (EGFR-TKI) is a first-line treatment option for patients with advanced NSCLC harboring EGFR mutations [2, 3], and many patients show clinical remission or disease control following treatment. However, after the initial response, most patients eventually experience disease progression.

Recent studies have reported on the mechanisms of drug resistance to EGFR-TKIs [4], and the most commonly identified is an additional single base substitution, T790 M, of the EGFR gene. Several rare resistant mutations, such as MET gene amplification or PTEN gene deletion, have also been identified [5]. Based on these resistant mechanisms, new and irreversible EGFR-TKIs have been found to be clinically effective in inhibiting the growth of NSCLC tumors with resistance to earlier EGFR-TKIs [6].

To facilitate new aspects of disease understanding and management, reassessing the tumor tissue at the time of disease progression is necessary to identify the mutational status of the acquired resistance [7]. To estimate the feasibility of re-biopsy in each patient, the possibility of obtaining sufficient tissue samples from relapsing lesions that re-grow or newly appear at the time of disease progression should be evaluated. However, in the clinical setting, re-biopsy can prove challenging in certain instances. Although recent reports have addressed the importance of re-biopsy, the mechanisms underlying the development of relapsing lesions and the possibility of re-biopsy of lesions in patients with resistance to EGFR-TKI remain unclear.

In the current study, we retrospectively investigated the patterns of disease progression and the feasibility of re-biopsy of targeted relapsing tumor lesions following the development of EGFR-TKI resistance in patients with NSCLC.

## Methods

### Patients

A total of 171 patients with NSCLC and EGFR gene mutation began EGFR-TKI treatment from 2008 to 2014 at our institute. Among these, 69 patients showed disease remission and subsequent disease re-progression, despite continuing EGFR-TKI treatment. The feasibility of re-biopsy for relapsing disease was evaluated in all 69 patients. Approval for this study was granted by the institutional review board (Fujita-HM16–283).

### Evaluation

Data were collected from patients’ medical records and a radiological image database, and two separate time points of relapsing disease were determined. The Response Evaluation Criteria In Solid Tumors (RECIST)-progressive disease (PD) time point was determined based on RECIST ver. 1.1 [8]. The clinical progressive disease (clinical-PD) time point was determined when the attending physician judged the lung cancer lesions to have progressed and the patient ceased EGFR-TKI therapy. The PD lesion (the lesion that led to the judgment of disease progression) was identified in the radiological images from both the RECIST-PD and clinical-PD time points. The size and site of PD lesions were investigated, and each lesion was evaluated for the feasibility of re-biopsy (accessibility and invasiveness of assumed biopsy methods) according to the criteria described below and in Table 1. When more than two lesions were identified as relapsing in a patient, all lesions were subsequently evaluated. Among these lesions, the most easily accessible lesion for biopsy was considered the “dominant lesion” and included in further analyses.

**Table 1:** Criteria to evaluate the feasibility of biopsy for the progressive lesions

Feasibility of biopsy	Criteria
Category A Accessible using conventional techniques	Lung nodules of long diameter ≥ 2 cm and positive CT bronchus sign^a^ for TBB Pulmonary infiltration (lymphangitis) for TBLB
	Intrapulmonary lesions of long diameter ≥ 1 cm and ≤5 cm distant from the thoracic wall for CTNB
	Hilar or mediastinal lymph node (#2, 4, 7, 8, 10, 11, 12) enlargement with a long diameter ≥ 1 cm for EBUS-TBNA (EUS-FNA)
	Pleural effusion with a mean thickness ≥ 1 cm on chest CT image for thoracentesis
	Peripheral lymph node or cutaneous lesions of long diameter ≥ 2 cm for needle biopsy Bone metastasis with invasion to surrounding soft tissue for CTNB
Category B Invasive or difficult (but possible) to biopsy	Lung nodules of long diameter 1 ≤ and <2 cm with positive CT bronchus sign for TBB
	Intrapulmonary lesions of long diameter < 1 cm and ≤5 cm distant from the thoracic wall, or lesions of long diameter ≥ 1 cm and >5 cm distant from the thoracic wall for CTNB Hilar or mediastinal lymph node #2, 4, 7, 8, 10, 11, 12) enlargement of long diameter < 1 cm for EBUS-TBNA (EUS-FNA)
	Pleural effusion with mean thickness < 1 cm on chest CT image for thoracentesis
	Intra-abdominal lesions (including hepatic or adrenal metastases) of long diameter ≥ 2 cm for CTNB Peripheral lymph node or cutaneous lesions of long diameter < 2 cm for needle biopsy
Category C Extremely difficult to biopsy or highly invasive	Lung nodules of long diameter < 1 cm or negative CT bronchus for TBB Intrapulmonary lesions of long diameter < 1 cm and >5 cm distant from the thoracic wall for CTNB Mediastinal lymph node accessible only by surgical mediastinoscopy or thoracoscopy Intra-osseous lesions (bone metastasis) Intracranial lesions and meningeal carcinomatosis Any lesions anatomically accessible only by surgical procedures

^a^CT bronchus sign defined according to the relationship between the target lesion and nearest bronchus [9]

*TBB* transbronchial biopsy, *TBLB* transbronchial lung biopsy, *CTNB* CT-guided needle biopsy, *EBUS-TBNA* endobronchial ultrasound guided transbronchial needle aspiration, *EUS-FNA* endoscopic ultrasound fine needle aspiration, *US* ultrasound

### Criteria for the feasibility of re-biopsy

Two pulmonologists (S.U. and K.I., with 10 and 25 years of experience in thoracic imaging, respectively) evaluated whether re-biopsy was feasible by reviewing the images from both the RECIST-PD and clinical-PD time points. The modality of re-biopsy (trans-bronchial lung biopsy [TBB], CT-guided needle biopsy [CTNB], or surgical biopsy) was selected virtually, according to the size and location of the lesions. The technical difficulty of the selected modality and the invasiveness to patients were assessed for each PD lesion and categorized into one of three categories: category A, accessible with conventional techniques; category B, difficult (but possible) to biopsy and accessible with invasive methods; and category C, extremely difficult to biopsy or inaccessible without using highly invasive methods. In this study, no PD lesion was identified for which an endobronchial bronchoscopic re-biopsy would be most suitable.

**Fig. 1:**
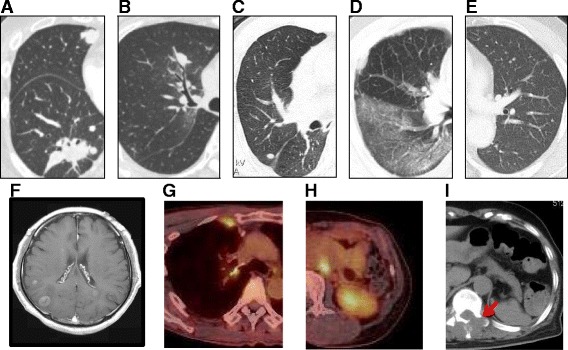
Representative cases for the assessment of re-biopsy feasibility Progressive or relapsing sites were evaluated for the feasibility of re-biopsy according to the criteria shown in Table 1. **a** Lung nodule (long diameter ≥ 2 cm) with a responsive bronchus leading to the center of the lesion (category A for trans-bronchial biopsy [TBB]). **b** Lung nodule adjacent to the nearest bronchus (category A for TBB). **c** Lung nodule not reaching a responsive bronchus but accessible by CT-guided needle biopsy (CTNB) (long diameter < 1 cm and ≤5 cm distant from the thoracic wall category B for CTNB). **d** Diffuse pulmonary infiltration presenting a lymphangitic distribution (category A for trans-bronchial lung biopsy [TBLB]). **e** Small pulmonary metastases (not feasible or extremely difficult for TBB or CTNB; category C). **f** Brain metastases (surgical resection required; category C). **g** Solitary pleural metastasis (long diameter ≤ 1 cm; category B for CTNB). **h** Para-aortic lymph node metastasis, front and adjacent to the abdominal aorta (CTNB is highly unsafe; category C). **i** Bone metastasis with extra-osseous invasion (category A for CTNB)

The feasibility of TBB re-biopsy for intra-pulmonary lesions was estimated according to the lesion size and computed tomography (CT) bronchus sign, which was determined by the relationship between the target lesion and the nearest bronchus, as described by Minezawa et al. [9]. Lesions with a positive CT bronchus sign (bronchus leading to the target lesion could be identified in a thin-slice chest CT) and diameter ≥ 2 cm were categorized as category A (represented in Fig. 1a-c), as we previously reported that the diagnostic yield with TBB when using endobronchial ultrasound with a guide sheath system for these lesions could be more than 80% [9]. Lesions that had a negative CT bronchus sign or were <1 cm in diameter were categorized as category C for TBB (Fig. 1e).

The feasibility of using CTNB for pulmonary lesions was determined according to the lesion’s size and distance from the chest wall. As previously reported, the diagnostic yield of CTNB for lesions with a diameter < 1 cm would be significantly decreased [10]. Thus, lesions that had a diameter ≥ 1 cm and were ≤5 cm distant from the chest wall were categorized as category A (Fig. 1g). In addition, as CTNB for lesions distant from the chest wall might be possible but difficult and might have a greater risk of pneumothorax complication, lesions that were located >5 cm from the nearest chest wall were categorized as category B for CTNB.

The feasibility of endobronchial ultrasound-guided transbronchial needle aspiration (EBUS-TBNA) for mediastinal or hilar lymph nodes was determined by their size and location (lymph node station). Enlarged lymph nodes (long diameter ≥ 1 cm) at #2, #4, #7, #8, #10, #11, or #12 stations were categorized as category A for EBUS-TBNA (#8 for endoscopic ultrasound-fine needle aspiration (EUS-FNA)). However, lymph nodes that were unreachable by EBUS-TBNA or EUS-FNA (#3, #5, #6, #9 stations) were categorized as category C, as surgical mediastinoscopy or thoracoscopy would be required to biopsy these lymph nodes. Pleural effusion showing a mean thickness ≥ 1 cm on chest CT images was categorized as category A based on a previous report indicating that effusions thicker than 1 cm on radiography are sufficiently large for sampling by thoracentesis [11]. Peripheral metastatic lesions (peripheral lymph nodes or cutaneous metastases) were also categorized into category A for needle biopsy. Intracranial lesions (brain metastases) were classified into category C, as biopsies on brain metastases typically require surgical procedures (Fig. 1e). Lumbar punctures for malignant meningitis were also categorized as category C because of the associated low sensitivity of disease diagnosis [12, 13]. CTNB for intra-osseous lesions was categorized as category C because such samples require processing by decalcification, which may damage the DNA for further analyses. However, bone metastases accompanied by invasion of the surrounding soft tissue were categorized as category A (Fig. 1i). Other metastatic lesions accessible only by surgical procedures were also categorized as category C (Fig. 1h).

According to these criteria, we evaluated all relapsing lesions of the 69 patients with EGFR-TKI-resistant NSCLC and determined the feasibility of re-biopsy for each, according to categories A, B, or C. If a patient had two or more relapsing lesions with different feasibility categories, the highest category (A > B > C) was applied.

### Statistical analysis

Statistical analyses were performed using EZR (Saitama Medical Center, Jichi Medical University, Saitama, Japan), a graphical user interface for R (The R Foundation for Statistical Computing, Vienna, Austria) (http://www.jichi.ac.jp/saitama-sct/SaitamaHP.files/statmedEN.html) [14]. Progression-free survival, overall survival, and the time interval between the RECIST-PD and clinical-PD were estimated using the Kaplan–Meier method. McNemar’s chi-squared test was used to evaluate the differences in feasibility between the two PD time points. Values of *p* < 0.05 were considered statistically significant.

## Results

During the study period, 69 NSCLC patients were identified as having acquired resistance to EGFR-TKI therapy after previous tumor shrinkage. Patient characteristics are shown in Table 2. The median age was 66 years (range 40–88), and 62% of the patients were women. All cases were, pathologically, adenocarcinomas, except for one pleomorphic carcinoma, and all were EGFR-mutation positive. The median progression-free survival was 13.9 months (95% confidence interval [CI], 5.5–22.6 months), and the median overall survival was 20.0 months (95% CI, 7.9–32.1 months). We analyzed the relapse sites and feasibility of re-biopsy in each case.

**Table 2:** Patient characteristics

Total number of patients	69
Age (years)	
Median (range)	66 (40–88)
Gender	
Male	26 (38%)
Female	43 (62%)
ECOG performance status (at the start of EGFR-TKI^a^)	
0	34 (49%)
1	17 (25%)
2	17 (25%)
≥ 3	1 (1%)
Pathologic diagnosis	
Adenocarcinoma	68 (99%)
Pleomorphic carcinoma	1 (1%)
Stage^a^ (at initial diagnosis)	
I	4 (6%)
II	4 (6%)
III	9 (13%)
IV	52 (75%)
EGFR mutation	
Exon 19 deletion	33 (48%)
Exon 21 L858R	31 (45%)
Other	5 (7%)
EGFR-TKI treatment	
Gefitinib	57 (83%)
Erlotinib	12 (17%)
Line of EGFR-TKI therapy	
First line	37 (53%)
Second line	19 (29%)
Third line or greater	13 (18%)

^a^Staging procedures were carried out using the 7th edition of the American Joint Committee on Cancer (AJCC) Cancer Staging Manual

### Relapse or relapsing sites after EGFR-TKI treatment failure

**Fig. 2:**
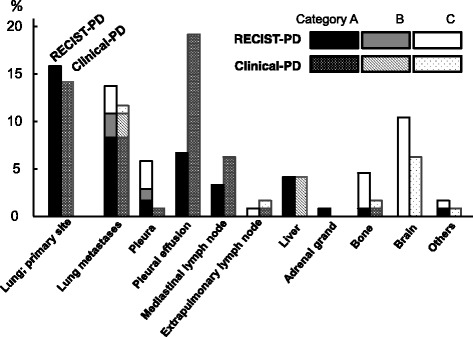
Distribution of dominant (most accessible) relapse sites and feasibility of re-biopsy of each lesion at RECIST-PD and clinical-PD In the 69 patients analyzed, the most common estimated re-biopsy target site at RECIST-PD was the re-grown primary tumor (lung), the second most common was intra-pulmonary metastasis, and the third was brain metastasis. At clinical-PD, the most frequent lesion was pleural effusion followed by primary lung lesions, intra-pulmonary metastases and brain metastases. The feasibility of re-biopsy was evaluated according to the criteria presented in Table 1

We analyzed all relapsing lesions in each patient at the RECIST-PD and clinical-PD time points and selected the most easily accessible lesion for re-biopsy as the estimated target site (Fig. 2). The most common estimated re-biopsy target site was the re-grown primary lung tumor at the time of RECIST-PD. The second most common site was intra-pulmonary metastasis, and the third was brain metastasis. At the clinical-PD time point, the most common lesion was pleural effusion, followed by primary lung lesion, intra-pulmonary metastasis, and brain metastasis. The median time interval between the RECIST-PD and the clinical-PD was 38 days (95%CI, 12–82 days).

### Feasibility of re-biopsy of relapsed lesions

The feasibility of re-biopsy of the most accessible relapsing lesion (lesions with the highest category; A > B > C [Table 1]) was assessed for each patient at RECIST-PD and clinical-PD (Fig. 2). At both the RECIST-PD and clinical-PD time points, relapsing lesions were frequently distributed in intrapulmonary lesions, including both primary and metastatic lesions. Among the intrapulmonary relapsing lesions, all those at primary sites were evaluated as category A (accessible with conventional techniques), whereas those at metastatic sites were evaluated as A, B (accessible with invasive methods or difficult [but possible] to biopsy), or C (inaccessible without highly invasive methods or extremely difficult to biopsy) (Table 1). All cases with pleural effusion, which was the most frequent relapse site at the clinical-PD time point, were categorized as category A. Brain metastases, which were the third and fourth most common relapse sites at the RESIST-PD and clinical-PD time points, respectively, were categorized as category C. Most bone metastases were evaluated as category C. Relapses with abdominal lesions, including liver and adrenal metastases, were evaluated as category B. Mediastinal lymph node relapses were accessible by EBUS-TBNA; thus, all such cases were categorized as category A.

### Comparison of re-biopsy feasibility between RESIST-PD and clinical-PD

**Fig. 3:**
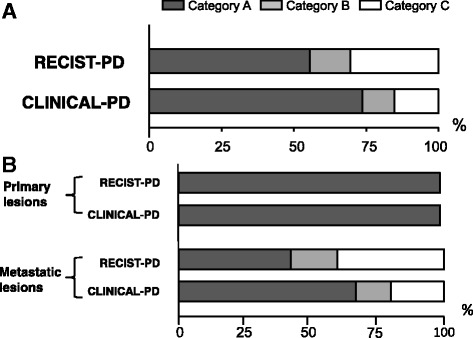
**a** Total proportion of re-biopsy feasibility at RECIST-PD and clinical-PD Re-biopsy feasibility may increase with disease progression. At RECIST-PD, 55, 13, and 32% of lesions were category **A**, **B**, and **C**, respectively. At clinical-PD, 74, 10, and 16% were category **A**, **B**, and **C**, respectively. **b** Comparison of re-biopsy feasibility at primary and metastatic lesions Re-biopsy for primary lesions was evaluated as category A in all cases, both at RECIST-PD and clinical-PD. However, the feasibility of re-biopsy of relapsed lesions in metastatic disease varies among patients and re-biopsy becomes less complex as progressive or relapsed lesions develop in the clinical course. Re-biopsy may be highly invasive or complex (category C) in some patients with metastatic relapse, even at clinical-PD

The total feasibility of re-biopsy is shown in Fig. 3a. At the RECIST-PD time point, the re-biopsy feasibility for the category A, B, and C relapse sites was 55, 13, and 32, respectively. At the clinical-PD time point, the feasibility of categories A, B, and C was 74, 10 and 16%, respectively. The total feasibility rate of re-biopsy (category A or B) at the clinical-PD was 84% and was significantly higher than that at the RECIST-PD, which was 68% (*p* = 0.0389). As expected, these data indicated that the feasibility of re-biopsy might increase with disease progression.

Notably, the relapsing lesion type with the highest feasibility category was metastatic lesions (77% of cases at RECIST-PD and 80% at clinical-PD). This finding indicates that re-biopsy samples can be obtained mainly from metastatic lesions in the clinical setting. Furthermore, the estimated biopsy feasibility from metastatic lesions was lower than that from regrowth lesions at primary sites. As shown in Fig. 3b, the feasibility of re-biopsy for primary lesions was evaluated as category A in all cases at both the RECIST-PD and clinical-PD time points. However, the feasibility of re-biopsy for metastatic lesions was evaluated as 42/17/42% for categories A/B/C, respectively, at RECIST-PD, and as 66/13/20% for categories A/B/C, respectively, at clinical-PD. This finding suggests that the feasibility of re-biopsy for relapsing lesions in metastatic disease varies among patients and that re-biopsy becomes less complex as progressive or relapsing lesions develop over the clinical course. However, our data also indicate the difficulty and invasiveness of performing re-biopsy in some patients, even at the clinical-PD time point.

## Discussion

In treatment strategies based on recent developments in mutation research in NSCLC, re-biopsy has become essential for investigating the tolerance mechanism of a progressed tumor after the initial TKI therapy [15]. At present, however, re-biopsy is performed only in selected cases in which disease lesions are easily accessible using conventional methods of biopsy [16, 17]. It is not clear whether re-biopsy could be applied as a standard technique in all cases of disease progression after chemotherapy. Few reports have evaluated the feasibility of re-biopsy at the time of PD in the clinical setting [18, 19]. In this study, data from all patients with relapsing disease after EGFR-TKI therapy at our institute were evaluated for the feasibility of re-biopsy by determining definite feasibility criteria for re-biopsy. We found a total feasibility of re-biopsy of 68% at the RECIST-PD time point and 84% at the clinical-PD time point. Although these percentages are relatively high, our study clearly indicates that a certain number of patients exist in whom re-biopsy at the time of PD would be extremely difficult or overly invasive. This finding may represent a critical disadvantage for the affected patients. The development of a robust and non-invasive re-biopsy method is therefore of importance [20, 21].

Our study also demonstrated that the feasibility of re-biopsy depends on the anatomical site and the size of the relapsed lesion. Re-biopsy is less complex if the tumor relapses as a pleural effusion or does so in a primary tumor growth of a certain size. Yoon et al. (2012) reported ineligible factors for biopsy by CT-guided lung biopsy that depended on the anatomical situation of the lesion, such as adjacency to a large central bronchus or vessels and miliary or diffuse distribution [22]. In cases with intracranial metastases, re-biopsy would be highly invasive and often impossible for older or disabled patients. It is also difficult to obtain high-quality DNA samples from intraosseous lesions because biopsy specimens from such lesions require processing by decalcification. Thus, if an intracranial or intraosseous lesion was the only relapsing site at PD, the re-biopsy feasibility was categorized as category C in our study. As previously reported, the anatomical site of the relapsing lesion is one of the major limitations to re-biopsy [23]. The retrospective study of Kawamura et al. [19] on the re-biopsy of PD cases in the clinical setting indicated that a patient’s disease condition can influence the physician’s decision to perform a re-biopsy [19]. Thus, our theoretically evaluated results should be interpreted carefully in accordance with patient factors in routine clinical settings.

Our study demonstrated that the feasibility of re-biopsy at clinical-PD was significantly increased compared with that at RESIST-PD, indicating that the feasibility of re-biopsy may increase over the patient’s clinical course. The timing of re-biopsy is a crucial consideration in the management of patients with EGFR-TKI. Our data might therefore be useful information for oncologists who are treating patients with lung cancer.

We also showed that the distribution of the estimated target site for re-biopsy varied between the RECIST-PD and clinical-PD time points, such that the frequency of mediastinal lymph nodes and pulmonary effusion increased and the frequency of intracranial and intraosseous lesions decreased at clinical-PD (Figs. 2 and 3). This finding indicates that along with disease progression, relapsing lesions became more diffuse and that re-biopsy could be performed more easily. Treatment options after EGFR-TKI therapy include treatment continuation with the same EGFR-TKI (“beyond-PD” treatment) [24, 25], switching to cytotoxic chemotherapy [26–29] or to immunotherapy (with anti-PD-1 or anti-PD-L1 antibodies, for example) [30] or the administration of third-generation EGFR-TKI agents such as osimertinib for relapsing tumors harboring a T790 M-resistant mutation [3, 31]. As the selection of an appropriate therapy based on molecular or immunohistological analyses at the turning point of disease progression is of critical importance, clinicians may occasionally wait for apparent disease progression before selecting the most easily accessible re-biopsy sites. Thus, the re-biopsy feasibility analyses at RECIST-PD and clinical-PD presented in this study might be beneficial for the management of tumors harboring EGFR mutations.

This study has several limitations. First, it is a retrospective analysis based on image data that were acquired in a routine clinical setting. Therefore, a certain degree of time lag between the estimated PD and the actual PD may have existed. If asymptomatic metastatic lesions were present, detection by imaging may have been delayed. However, in most cases, body CT imaging had been obtained at least every 6 months, and brain CT or MRI had been obtained at intervals of at least 1 year. Second, the feasibility of re-biopsy was estimated virtually by analyzing the size and position of relapsing lesions, mainly from the radiological findings. Although our analyses were partly based on those used in a previous study [10], the success rate of re-biopsy may depend on various conditions, including the technical skill available to perform each biopsy method at a given institution or the patient’s condition and consent for re-biopsy. In addition, not all biopsy specimens might be suitable for mutation or immunohistological analyses. Previous reports have shown that the technical success rate for re-biopsy using CT-guided needle biopsy was 100% in cases selected by rigorous imaging criteria, but that the success rate for mutation analysis in the acquired specimen was approximately 80% [23]. Another report showed that the success rate for re-biopsy using any method was 82%, with 25.6% producing insufficient tumor cells for mutation analyses [32]. Considering these factors, the actual possibility of re-biopsy may be lower in the clinical setting than has been estimated. Following the approval of osimertinib in Japan, we treated 31 patients with relapsing disease after first-line EGFR-TKI therapy at our institute. Twenty-four patients (77.4%) underwent re-biopsy (6 patients at the RECIST-PD and 18 patients at the clinical-PD). All lesions for re-biopsy were evaluated as category A according to our interpretation (Table 1). Seven patients (22.6%) did not undergo re-biopsy because the relapsed lesion was evaluated as category C in 5 patients (2 were meningeal lesions and 3 were brain metastases), and the general condition of the remaining 2 patients was too deteriorated for re-biopsy. Among all of the samples obtained by re-biopsy, 82.5% were eligible for molecular analysis. Despite the small scale of our data, our study results suggest that the analysis of image data is comparable to real-world data, although it is important to consider the biopsy sample quality and patient condition in a real-world re-biopsy. Therefore, to interpret the feasibility of re-biopsy more precisely, a prospective study is needed. In addition, the development of more robust and reliable and less invasive methods for mutation analyses would be of significant benefit.

## Conclusions

The feasibility of re-biopsy for relapsing disease among NSCLC patients with acquired resistance to EGFR-TKI was evaluated. Although re-biopsy for relapsing disease may be feasible in many cases, prospective data are required on the outcomes of the procedures in terms of molecular data. Re-biopsy might be highly invasive or extremely difficult in certain patients, especially at the RECIST-PD time point. To facilitate an appropriate treatment strategy for NSCLC patients, the standardization of re-biopsy by reliable methods is essential.

## Abbreviations

Clinical-PD: Clinical progressive disease
CT: Computed tomography
CTNB: CT-guided needle biopsy
EBUS-TBNA: Endobronchial ultrasound-guided transbronchial needle aspiration
EGFR: Epidermal growth factor receptor
EGFR-TKI: EGFR tyrosine kinase inhibitor
EUS-FNA: Endoscopic ultrasound-fine needle aspiration
NSCLC: Non-small cell lung cancer
PD: Progressive disease
RECIST: The Response evaluation criteria in solid tumors
RECIST-PD: RECIST-progressive disease
TBB: Trans-bronchial lung biopsy
TKI: Tyrosine kinase inhibitor

## References

[1] Miller KD, Siegel RL, Lin CC, Mariotto AB, Kramer JL, Rowland JH (2016). Cancer treatment and survivorship statistics. CA Cancer J Clin.

[2] Mok TS, Wu YL, Thongprasert S, Yang CH, Chu DT, Saijo N (2009). Gefitinib or carboplatin-paclitaxel in pulmonary adenocarcinoma. N Engl J Med.

[3] Maemond M, Inoue A, Kobayashi K, Sugawara S, Oizumi S, Isobe H (2010). Gefitinib or chemotherapy for non-small- cell lung cancer with mutated EGFR. N Engl J Med.

[4] Shea M, Costa DB, Rangachari D (2016). Management of advanced non-small cell lung cancers with known mutations or rearraengements: latest evidence and treatment approaches. Ther Adv Resp Dis.

[5] Kobayashi Y, Mitsudomi T (2016). Not all EGFR mutations in lung cancer are created equal: perspectives for individualized treatment strategy. Cancer Sci.

[6] Jänne PA, Yang JC, Kim DW, Planchard D, Ohe Y, Ramalingam SS (2015). AZD9291 in EGFR inhibitor-resistant non-small cell lung cancer. New Engl J Med.

[7] Jekunen AP. Role of rebiopsy in relapsed non-small cell lung cancer for directing oncology treatment. J Oncol. 2015;2015:809835. 10.1155/2015/809835 PMC4325200 25699082

[8] Eisenhauer EA, Therasse P, Bogaerts J, Schwartz LH, Sargent D, Ford R (2009). New response evaluation criteria in solid tumours: revised RECIST guideline (version 1.1). Eur J Cancer.

[9] Minezawa T, Okamura T, Yatsuya H, Yamamoto N, Morikawa S, Yamaguchi T (2015). Bronchus sign on thin-section computed tomography is a powerful predictive factor for successful transbronchial ultrasound with a guide sheath for small peripheral lung lesions: a retrospective observational study. BMC Med Imag.

[10] Shimizu K, Ikeda N, Tsuboi M, Hirano T, Kato H (2006). Percutaneous CT-guided fine needle aspiration for lung cancer smaller than 2 cm and revealed by ground-glass opacity at CT. Lung Cancer.

[11] Daniels CE, Ryu JH (2011). Improving the safety of thoracentesis. Curr Opin Pulm Med.

[12] Clarke JL, Perez HR, Jacks LM, Panageas KS, Deangelis LM (2010). Leptomeningeal metastases in the MRI era. Neurology.

[13] Straathof CS, de Bruin HG, Dippel DW, Vecht CJ (1999). The diagnostic accuracy of magnetic resonance imaging and cerebrospinal fluid cytology in leptomeningeal metastasis. J Neurol.

[14] Kanda Y (2013). Investigation of freely available easy-to-use software ‘EZR’ for medical statistics. Bone Marrow Transplant.

[15] Eberhard DA, Giaccone G, Johnson BE (2008). Biomarkers of response to epidermal growth factor receptor inhibitors in non-small-cell lung cancer working group: standardization for use in the clinical trial setting. J Clin Oncol.

[16] Ishii H, Azuma K, Yamada K, Matsuo N, Nakamura M (2017). Accuracy of transbronchial biopsy as a rebiopsy method for patients with relapse of advanced non-small-cell lung cancer after systemic chemotherapy. BMJ Open Resp Res.

[17] Hata A, Katakami N, Nanjo S, Okuda C, Kaji R (2017). Rebiopsy of histological samples in pretreated non-small cell lung cancer: comparison among rebiopsy procedures. In Vivo.

[18] Kim L, Tsao MS (2014). Tumor tissue sampling for lung cancer management in the era of personalized therapy: what is good enough for molecular testing?. Eur Respir J.

[19] Kawamura T, Kenmotsu H, Taira T, Omori S, Nakashima K (2016). Rebiopsy for patients with non-small-cell lung cancer after epidermal growth factor receptor-tyrosine kinase inhibitor failure. Cancer Sci.

[20] Thress KS, Brant R, Carr TH, Dearden S, Jenkins S (2015). 2015. EGFR mutation detection in ctDNA from NSCLC patient plasma: a cross-platform comparison of leading technologies to support the clinical development of AZD9291. Lung Cancer.

[21] Nishio M, Goto K, Chikamori K, Hida T, Katakami N, Maemondo M (2016). 2016. Analysis of epidermal growth factor receptor mutations in serum among Japanese patients treated with first-line erlotinib for advanced non-small-cell lung cancer. Clin Lung Cancer.

[22] Yoon HJ, Lee HY, Lee KS, Choi YL, Ahn MJ, Park K (2012). Repeat biopsy for mutational analysis of non-small cell lung cancers resistant to previous chemotherapy; adequacy and complications. Radiology.

[23] Hasegawa T, Sawa T, Futamura Y, Horiba A, Ishiguro T, Marui T (2015). 2015. Feasibility of rebiopsy in non-small cell lung cancer treated with epidermal growth factor receptor-tyrosine kinase inhibitors. Inter Med.

[24] Chen Q, Quan Q, Ding L, Hong X, Zhou N, Liang Y (2015). Continuation of epidermal growth factor tyrosine kinase inhibitor treatment prolongs disease control in non-small-cell lung cancers with acquired resistance to EGFR tyrosine kinase inhibitors. Oncotarget.

[25] Soria JC, Wu YL, Nakagawa K, Kim SW, Yang JJ, Ahn MJ (2015). 2015. Gefitinib plus chemotherapy versus placebo plus chemotherapy in EGFR-mutation-positive non-small-cell lung cancer after progression on first-line gefitinib (IMPRESS): a phase 3 randomised trial. Lancet Oncol.

[26] Kanda S, Horinouchi S, Fujiwara Y, Nokihara H, Yamamoto N, Sekine I (2015). 2015. Cytotoxic chemotherapy may overcome the development of acquired resistance to epidermal growth factor receptor tyrosine kinase inhibitors (EGFR-TKIs) therapy. Lung Cancer.

[27] Miyauchi E, Inoue A, Kobayashi K, Maemondo M, Sugawara S, Oizumi S (2015). Efficacy of chemotherapy after first-line gefitinib therapy in EGFR mutation-positive advanced non-small cell lung cancer – data from a randomized phase III study comparing gefitinib with carboplatin plus paclitaxel (NEJ002). Jpn J Clin Oncol.

[28] Matsuda T, Imai H, Kuwako T, Miura Y, Yoshino R, Kaira K (2015). Efficacy of platinum combination chemotherapy after first-line gefitinib treatment in non-small cell lung cancer patients harboring sensitive EGFR mutations. Clin Transl Oncol.

[29] Topalian SL, Hodi FS, Brahmer JR, Gettinger SN, Smith DC, McDermott DF (2012). 2012. Safety, activity, and immune correlates of anti–PD-1 antibody in cancer. N Engl J Med.

[30] Sequist LV, Soria JC, Goldman JW, Wakelee HA, Gadgeel SM, Varga A (2015). Rociletinib in EGFR-mutated non–small-cell lung cancer. N Engl J Med.

[31] Califano R, Romanidou O, Mountzios G, Landi L, Cappuzzo F, Blackhall F (2016). Management of NSCLC disease progression after first-line EGFR tyrosine kinase inhibitors: what are the issues and potential therapies?. Drugs.

[32] Ashworth A, Rodrigues G, Boldt G, Palma D (2013). Is there an oligometastatic state in non-small dell lung cancer? A systematic review of the literature. Lung Cancer.

